# Molecular Characterization of Hemorrhagic Enteritis Virus (HEV) Obtained from Clinical Samples in Western Canada 2017–2018

**DOI:** 10.3390/v12090941

**Published:** 2020-08-26

**Authors:** Victor Palomino-Tapia, Darko Mitevski, Tom Inglis, Frank van der Meer, Mohamed Faizal Abdul-Careem

**Affiliations:** 1 Department of Ecosystem and Public Health, Faculty of Veterinary Medicine, University of Calgary, Health Research Innovation Center 2C53, 3330 Hospital Drive NW, Calgary, AB T2N 4N1, Canada; victor.palominotapia@ucalgary.ca (V.P.-T.); fjvander@ucalgary.ca (F.v.d.M.); 2 Poultry Health Services, 1-4 East Lake Ave NE, Airdrie, AB T4A 2G8, Canada; darko.mitevski@poultryhealth.ca; 3 The Institute of Applied Poultry Technologies, 201–151 East Lake Blvd, Airdrie, AB T4A 2G1, Canada; tom.inglis@poultryhealth.ca

**Keywords:** turkey hemorrhagic enteritis virus, immunosuppression, meat turkeys, molecular epidemiology, whole genome sequencing

## Abstract

Hemorrhagic enteritis virus (HEV) is an immunosuppressive adenovirus that causes an acute clinical disease characterized by hemorrhagic gastroenteritis in 4-week-old turkeys and older. Recurrent incidence of secondary infections (e.g., systemic bacterial infections, cellulitis, and elevated mortality), may be associated with the presence of field-type HEV in Canadian turkey farms. We speculate that field-type HEV and vaccine/vaccine-like strains can be differentiated through analysis of the viral genomes, hexon genes, and the specific virulence factors (e.g., ORF1, E3, and *fib* knob domain). Nine out of sixteen spleens obtained from cases suspected of immunosuppression by HEV were analyzed. The limited data obtained showed that: (1) field-type HEV circulates in many non-vaccinated western Canadian flocks; (2) field-type HEV circulates in vaccinated flocks with increased recurrent bacterial infections; and (3) the existence of novel point mutations in hexon, ORF1, E3, and specially *fib* knob domains. This is the first publication showing the circulation of wild-type HEV in HEV-vaccinated flocks in Western Canada, and the usefulness of a novel procedure that allows whole genome sequencing of HEV directly from spleens, without passaging in cell culture or passaging in vivo. Further studies focusing more samples are required to confirm our observations and investigate possible vaccination failure.

## 1. Introduction

Hemorrhagic enteritis virus (HEV) or *Turkey siadenovirus A*, a member of the family *Adenoviridae*, genus *Siadenovirus*, is a ubiquitous poultry pathogen. HEV has a linear, double-stranded DNA genome of 26.6 kilo base pairs (kb) [1] and codes for eight open reading frames (ORFs) distributed in two clusters [2]. Within these, the hexon and fiber proteins are important for their involvement in cell attachment and entry, plus the induction of neutralizing antibodies and protection against the disease [3,4,5]. HEV is the etiological agent of hemorrhagic enteritis (HE), a disease characterized by immunosuppression in turkeys of >4-weeks of age. The disease has two presentations: (1) clinical disease consisting of depression, gastrointestinal hemorrhages, and transient immunosuppression followed by increased mortality (up to 80% for highly virulent strains due to blood loss and secondary infection with opportunists like *Escherichia coli*) [6,7,8]; and (2) subclinical infection, consisting in immunosuppression and causing economical losses because of secondary bacterial infection, especially from *Escherichia coli*, and processing plant condemnations [7,9,10]. The immunosuppression caused by the subclinical infection increases the birds susceptibility to secondary bacterial infections which poses a problem for the judicious antibiotic use in farm animals, both being important problems for the turkey industry [11]. 

The rate of clinical disease (bloody feces and acute mortality) has become low due to vaccination and circulation of avirulent HE in the field [12], yet, many reports have suggested that avirulent strains are able to trigger subclinical infection in turkeys, causing strong immunosuppression and losses due to exacerbation of viral and bacterial diseases [7,13]. Despite this, some Canadian farmers do not regularly vaccinate their turkey flocks against HE due to the absence of clinical disease amidst seroconversion in the flocks, disregarding the potential immunosuppressive nature of these avirulent strains. Currently, HEV is immunosuppressive and responsible for morbidity and mortality [6,14,15]. 

Transmission of HEV can be horizontal through fecal-oral/cloacal routes [16,17,18,19] and, unlike other adenoviruses there is no evidence of vertical transmission [1,6], insect vectors are not known. Recent data suggests that recovered birds can become persistently infected and in some cases become long term virus shedders [20], in this way, contributing to the persistence of the pathogen in the population. Being an adenovirus, it is resistant when it is protected from drying [21,22], and it will remain viable for up to 7 weeks in contaminated carcasses or feces [1]. This environmental resistance contributes to the HEV survival despite activities such as cleaning, and disinfection in between production cycles. 

Upon ingestion or cloacal entry, the virus replicates in the gastrointestinal tract leading to a primary viremia from which the virus spreads to other internal organs, such as the bursa of Fabricius and spleen. As HEV is considered a lymphotropic and lymphocytopathic virus [23,24], it primarily targets immunoglobulin (Ig)M bearing B-lymphocytes in the bursa of Fabricius and spleen [25], notably, HEV targets macrophages [26]. Transient immunosuppression, characterized by reduced antibody production by B cells, and diminished phagocytosis activity by macrophages, becomes evident during acute phase of the infection [27,28]. At the same time, high levels of virus can be observed in the small intestine lamina propria together with intestinal congestion and hemorrhage, probably caused by the release of prostaglandins and histamine by mast cells [6,24]. This transient immunosuppressive effect will be more profound in HE caused by virulent strains with hemorrhagic enteritis; compared to avirulent strains [29,30]. However, avirulent strains are not apathogenic and could also cause immunosuppression [1,7,13]. It is known that pathogenic and apathogenic viral sequences may be differentiated using a process known as whole genome sequencing, which determines the complete nucleic acid sequence of an organism genome. This technique has become a useful tool for investigating the presence of virulence factors and epidemiological surveillance [31,32]. However, this process requires researchers to have a great concentration and proportion of viral DNA in the analyzed sample, which is usually obtained by viral propagation. There are limited options for propagating HEV as isolation mainly occur in: (1) naïve ≥6-week-old specific pathogen-free (SPF) turkeys which are scarce and difficult to obtain [33], and (2) the immortalized cell-line RP19 [34], which grows in suspension, requires extensive paperwork for its use, and may not work for all isolates. Thus, in this paper we propose a new method to study the virus by whole genome sequencing, without the need of passaging HEV in expensive/difficult systems.

HEV seems to have only one serotype, and research in the 70s showed that avirulent strains prevented clinical disease caused by virulent strains [35]. This led to the development of the Domermuth strain which is still used as a vaccine (splenic) in Europe. Cell-mediated role in the protection against clinical signs is not well understood [8,9,27,28]; however, maternal antibodies are important, as it is expected to find passive immunity in the progeny of vaccinated turkey breeders and to protect the poults for the first 2–3 weeks of life [8,36]. Currently, three types of vaccine are used in poultry operations worldwide: (a) live, commercial, or autogenous “splenic” vaccines, produced from spleens of HEV-infected SPF turkeys; (b) live, tissue culture derived vaccines, available in most countries and currently the only vaccine type available in Canada; and (c) inactivated vaccines, used more commonly in countries where no live vaccines are available [8,12]. Use of either live vaccine variants (a and b) will induce seroconversion and lead to protection against virus challenge [37], however, the splenic vaccines induce a strong and immediate immunity and can be given as emergency vaccine during an outbreak [8]. Because of this, splenic vaccines are regarded as a more potent vaccine compared to the tissue culture vaccine and requires less revaccinations in the field to achieve a protective antibody titer [8,36]. 

The Canadian turkey meat industry, with ~160 million kg of turkey meat in 2019 [38], is small in comparison with other countries, such as the United States (3.25 billion kg in 2019) [39]. This is of importance as the access to some vaccines, drugs, and ELISA kits is limited due to market constrains. In Canada, the tissue culture vaccine is the only vaccine approved for HEV control and is applied once, using a full dose (≥10^2.6^ TCID_50_) between 3.5–6 weeks of age, or twice, using a lower dose (e.g., 2/3 of a dose or ≥10^2.4^ TCID_50_) at days 25 and 35. This strategy is designed to reduce field HEV circulation in susceptible birds by immunizing birds with low maternal antibodies with the first vaccine delivery (day 25), and to infect those who were not immunized at the first vaccination due to high levels of neutralizing maternal antibodies or low vaccine intake (day 35). In addition, some farmers rely on circulation and protection generated by field avirulent strains and will, therefore, not vaccinate as there is no manifestation of clinical disease, overlooking the immunosuppressive potential of these field HEV viruses. 

Recently, several virulence factors of HEV were identified (i.e., hexon, open reading frame 1 (ORF1), E3, and *fib* knob domain) [12,40]. HEV variants containing these factors are circulating in vaccinated flocks leading to subclinical infections [12,41]. Our objective was to characterize these HEV-positive samples based on whole genome sequencing and/or gene sequences (i.e., hexon, ORF1, E3, *fib* knob domain) for determination of HEV origin. 

## 2. Materials and Methods 

### 2.1. Sample Collection, Processing, and Ultracentrifugation

**Figure 1:**
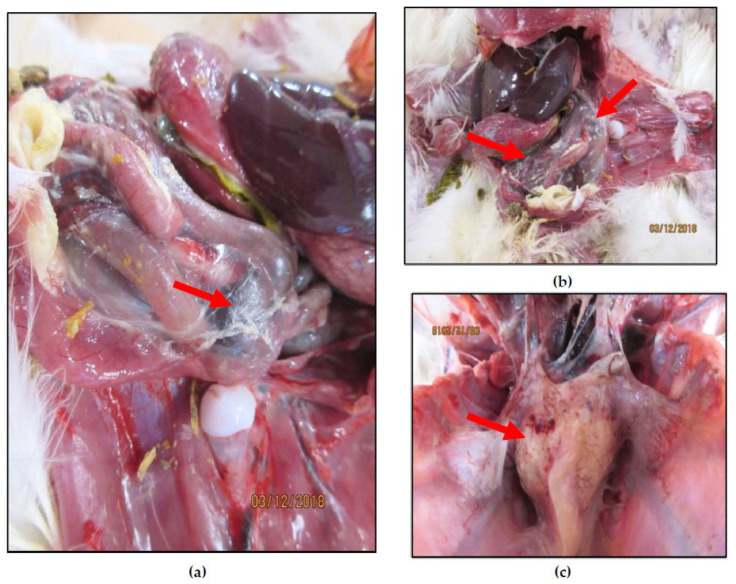
Systemic bacterial infection in 52-day-old turkeys (case 18-0374). This case was submitted due to elevated mortality in a flock without hemorrhagic enteritis virus (HEV) vaccination. The HEV sequence recovered from the spleen was found to be different from vaccine strains and to have missense mutations on the three virulence factors described by Beach et al. 2009 [40], and in the hexon protein. Airsacculitis lesions can be observed in (**a**,**b**); while pericarditis lesions can be observed in (**c**). *Escherichia coli* was isolated from pericardium and spleen.

Between July 2017–September 2018, a total of 16 spleen samples from Alberta (AB), British Columbia (BC), and Ontario (ON) were collected from turkey clinical cases submitted to Poultry Health Services (PHS) (Airdrie, AB, Canada), a private veterinary practice, by concerned growers with commercial turkey flocks experiencing increased mortality or secondary bacterial infections when compared with industry average, management guides, and/or current literature [42,43]. The clinical cases were characterized by cellulitis, systemic bacterial infection, and gangrenous dermatitis (Figure 1). Animals aged 44–117 days (average 76 days) were subjected to necropsy and sample collection at the post-mortem facility at the Veterinary Professional Centre (VPC) (Airdrie, AB, Canada).

Of the 16 spleen samples, 9 were HEV-positive by qPCR method [44], which was conducted at the Institute for Applied Poultry Technologies (IAPT). The positive samples were aliquoted in 1.5 mL tubes and stored at −80 °C until further processing. Moreover, samples from two commercially-available HE vaccines, namely Oralvax HE (Intervet Inc., Merck Animal Health, Omaha, NE, USA), and H.E. Vac (Arko Laboratories, LTD., Jewell, IA, USA) were obtained from PHS, aliquoted, and stored at −80 °C until further processing (Table 1). 

Spleen samples added to sterile tubes prefilled with 1.0 mm zirconium beads (Benchmark Scientific Sayreville, NJ, USA) on ice, and homogenized (BeadBug, Benchmark Scientific, Sayreville, NJ, USA) during three series of 30 s each at 300 RPM. Samples were kept on ice for 3 min in between series. Following disruption, the samples were centrifugated at 7500× *g* for 20 min at 4 °C and the supernatant filtered using a 0.2 µM syringe filter (Millipore Sigma, Burlington, MA, USA) and kept on ice for further processing. 

A purification method using ultracentrifugation technique with Optiprep as an iodixanol gradient was used to purify and concentrate HEV [45,46]. Briefly, the technique was adapted by adjusting the volumes for use with 3.3 mL ultracentrifuge tubes (Optiseal, Beckman Coulter, Fullerton, CA, USA). The highest concentration of virus genome [47] in relation of concentration of host genome [48] was detected by qPCR on phases 8 and 9 (area between 25% iodixanol and 40% iodixanol) and those were collected and processed for DNA extraction. 

### 2.2. DNA Extraction, PCR, and Sequencing 

Total DNA was extracted from reconstituted vaccine vials, and ultracentrifugation phases using a QIAamp DNA Mini Kit according to manufacturers’ instruction (Qiagen, Valencia, CA, USA). Invitrogen Platinum SuperFi PCR Master Mix (ThermoFisher Scientific, Waltham, MA, USA) was used for whole genome amplification, while Platinum Hot Start Taq PCR Master Mix (2×) (ThermoFisher Scientific, Waltham, MA, USA) was used for ORF1, E3, and *fib* knob gene amplification. Due to the high level of host DNA (nuclear and mitochondrial) in the spleen, low levels of HEV virus in persistently-infected spleens due to HEV seroconversion of the flock at the end of production [20], and multiplex Illumina sequencing, it was not possible to obtain the entire HEV genome without pre-amplification of the sample.

The entire HEV genome was amplified using the primers: THEV-Whole-F1, and THEV-Whole-R1 (Table 2) targeting conserved parts at the N and C terminus of the viral genome. The reaction consisted of 1 µM forward primer THEV-Whole-F1, 1 µM reverse primer THEV-Whole-R1, 12.5 µL 2× Platinum Superfi and 10 µL of DNA template for a total of 25 µL reaction mix. PCR thermocycler conditions consisted of opening denaturation (95 °C, 2 min) and 35 cycles of 95 °C for 10 s, 59 °C for 10 s, 68 °C for 14 min, and a terminal extension (68 °C, 5 min) resulting in a 26.1 kb amplicon. Following clean up (ExoSAP-IT Express PCR Product Cleanup, Applied Biosystems, Santa Clara, CA, USA) and quantification (Nanodrop 1000, ThermoScientific, Wilmington DE, USA) the DNA was submitted for next generation sequencing (NGS) using a Nextera XT library and the v3600 cartridge (MiSeq, Illumina, San Diego, CA, USA) at the Université de Montréal, QC, Canada. Sanger sequencing for ORF1, E3, and Fib genes using primers on Table 2, was used to confirm NGS data, and for the two sequences which render incomplete NGS (17-0495; and 18-0430).

Samples which rendered an incomplete whole genome sequencing were subjected to Sanger Sequencing of the ORF1, E3, and Fib genes. The ORF1 gene (1508 bp out of 1553 bp) was amplified using 1 set of primers (Alkie-HEV-ORF1-F1, and Alkie-HEV3’Rev) resulting in a 1508 bp amplicon. An additional primer was used for sequencing (HEV3’For-951) (Table 2). The reaction for ORF1, E3, and Fib consisted of 5 µM forward primer, 5 µM reverse primer, 12.5 µL 2x Master Mix, 7.5 µL of nuclease-free H2O, and 2.5 µL of DNA template for a total of 25 µL reaction mix. PCR thermocycler conditions consisted of initial denaturation (94 °C, 3 min) and 30 cycles of 94 °C for 30 s, annealing for 30 s, 72 °C for extension, and a final extension (68 °C, 7 min). Annealing temperatures and extension times specific to each primer pair can be found in Table 2. 

The PCR fragments were cleaned with E.Z.N.A. Gel Extraction Kit (Omega Bio-tek Inc., Norcross, GA, USA) and Sanger sequenced using primers depicted in Table 1 (University of Calgary, Core DNA services, Calgary, AB, Canada). Hexon gene comparisons were done only using sequences obtained using NGS (Table 3), both Sanger and NGS derived sequences where used for the analysis of the other genes. Reference strains used are shown in Table 3.

### 2.3. Data Analysis 

NGS short reads were mapped to the splenic vaccine strain (Dindoral SPF; Merial GmbH, Hallbergmoos, Germany) (GenBank accession #AY849321.1) [40,41] under App Map function on CLC Genomics Workbench v 12.0.2 (Qiagen, Valencia, CA, USA) using default settings, and complemented using Geneious assembler v10.2.6 (Biomatters LTD., Auckland, New Zealand) [49]. Whole genome sequences were aligned with MAFFT v7.450 [50,51], and phylogenetic trees were generated using Randomized Axelerated Maximum Likelihood (RAxML) v8.2.11 by applying the nucleotide model GTR+gamma [52]. Hexon, ORF1, E3, and *fib* knob domain nucleotide and amino acid alignments were performed using Clustal Omega v1.2.2., and phylogenetic trees were generated using RAxML applying the protein model BLOSUM62+gamma. All the sequences were deposited in GenBank (Table 3). *Fib* knob domain sequences were further evaluated using both the NetNGlyc (http://www.cbs.dtu.dk/services/NetNGlyc/) and NetOGlyc (http://www.cbs.dtu.dk/services/NetOGlyc/) online prediction services (DTU Bioinformatics, Department of Bio and Health Informatics, DTU Health Tech, Lyngby, Denmark). Structural locations of amino acid mutations on 3-D structure of published fowl adenovius-1 (FAdV-1) hexon protein (PDB code 2INY [53]), human adenovirus (HAdV) 2 and 5 (PDB codes 1P2Z, and 1P30 [54,55]), and HEV *fib* knob domain (PDB code 4CW8 [4]), were carried out using PYMOL v.4.6.0 (Shrödinger LLC, Cambridge MA, USA).

## 3. Results

### 3.1. Whole Genome Sequencing

**Figure 2:**
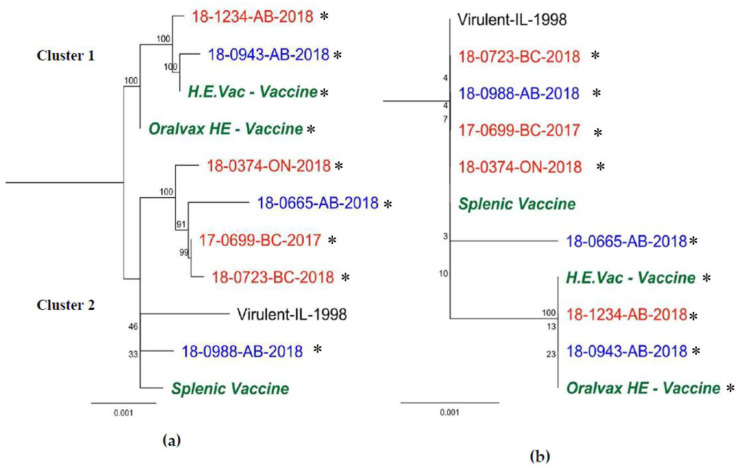
Nucleotide RAxML-based phylogenetic tree of complete HEV sequences (**a**); and amino acid RaxML-based phylogenetic trees of hexon gene (**b**), respectively. The included sequences are described in Table 3, and Supplement Table S1 Sequences in bold green are the vaccines sequences derived in the present study, bold red are sequences derived from non-vaccinated flocks, and bold blue from vaccinated flocks. Sequences obtained in this study are marked with a black asterisk. GenBank accession numbers and naming structure can be found at Table 3.

**Figure 3:**
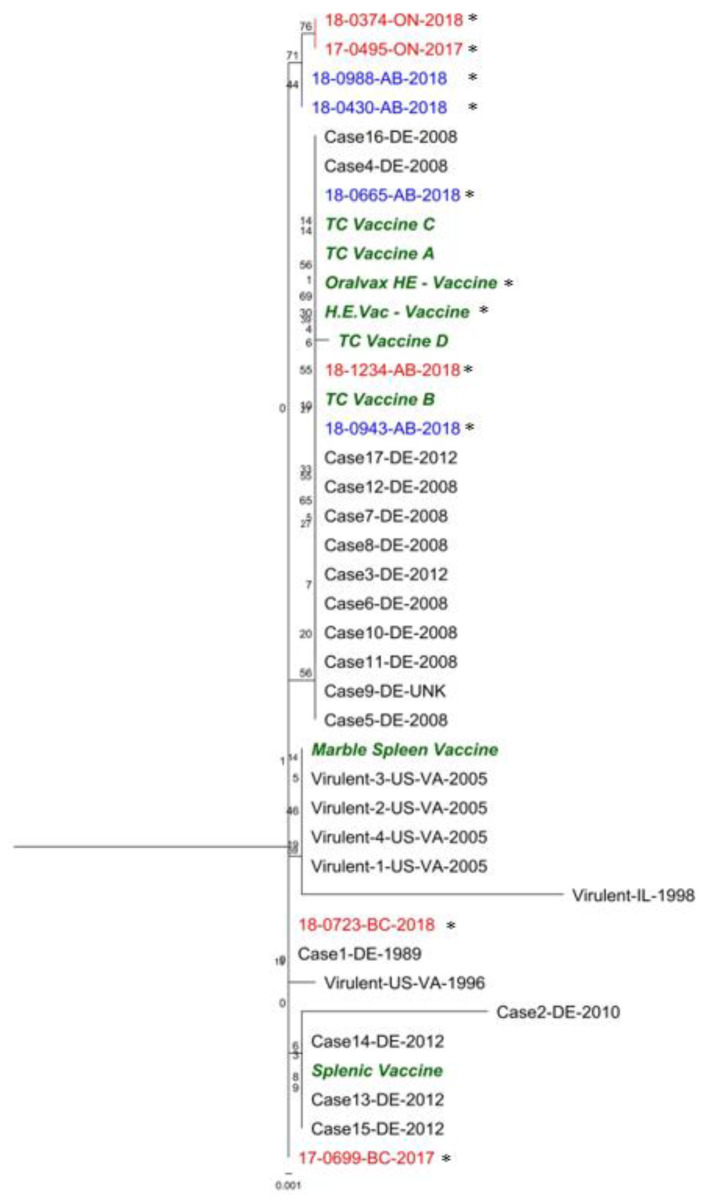
ORF1 maximum likelihood (ML) tree. Sequences shows all ORF1 HEV sequences previously published in GenBank, and those obtained in the present project. Sequences in bold green were obtained from vaccines in the present study, bold red from non-vaccinated flocks, and bold blue from vaccinated flocks. Sequences obtained in this study are marked with a black asterisk. GenBank accession numbers and naming structure can be found at Table 3.

**Figure 4:**
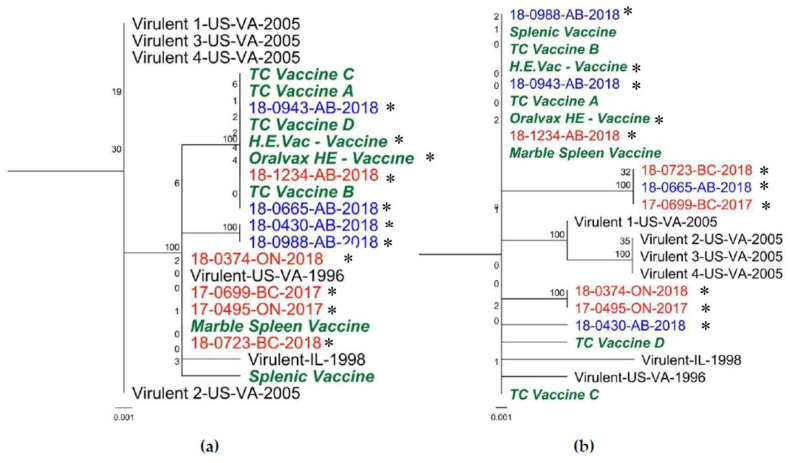
E3 (**a**) and *fib* knob domain (**b**) maximum likelihood (ML) trees. Sequences in bold green were obtained from vaccines in the present study, bold red from non-vaccinated flocks, and bold blue from vaccinated flocks. Sequences obtained in this study are marked with a black asterisk. GenBank accession numbers and naming structure can be found at Table 3.

The complete genome sequences of HEV positive samples with their respective GenBank number and publication are shown in Table 3, whereas genome size and classification are shown in Table 1. These sequences were grouped within two clusters. The first cluster included four sequences: Two commercial vaccines available in Canada (H.E.Vac, and Oralvax HE), and two HEV sequences 18-0943-AB-2018, and 18-1234-AB-2018; a second cluster included seven sequences distributed in two sub clusters, the first subcluster containing four sequences 18-0374-ON-2018, 18-0665-AB-2018, 17-0699-BC-2017, and 18-0723-BC-2018; and a second subcluster containing three sequences splenic vaccine, Virulent-IL-1998, and 18-0988-AB-2018 (Figure 2a). The following are the findings of the whole genome alignment when comparing the consensus sequence with each sequence: (1) 127 point-mutations; (2) a 3-bp change; (3) a 3-bp insertion; (4) a 2-bp change; (5) a 1-bp insertion (ORF1 Frameshift on Virulent-IL-1998); and (6) a 53-bp segment on a non-coding region showing great variability between strains with a 22-bp insertion in some strains. These changes resulted into 52 non-synonymous mutations in ORF1, IVa2, polymerase (AdPol), preterminal protein (pTP), pVII, hexon, DBP, 100K, 33K, E3, fiber (outside and inside the *Fib* domain), and ORF7 (Supplement Table S1). Upon amino acid alignment analysis of the whole genome sequences, amino acid differences in structural proteins were located in the hexon (two amino acid changes) and fiber (eight amino acid changes) proteins. Phylogenetic trees (Figure 2, Figure 3 and Figure 4) showed that most of the sequences differed from the vaccine sequences included in the analysis (seven out of nine). 

### 3.2. Hexon Gene 

Using the NGS sequences, thirteen single-point mutations were located in the hexon gene (2721 bp) of which 11 were silent. Two non-synonymous mutations consisted of a ntA231C (aaE77D) on 18-0665, and a ntG2598C (aaE866D) mutation in H.E. Vac, Oralvax HE and vaccine-like sequences 18-0943-AB-2018 and 18-1234-AB-2018 (Supplement Table S1) were sequenced in this study. The phylogenetic tree in Figure 2b clusters in one branch all commercial vaccines and the HEV sequences 18-0943-AB-2018, and 18-1234-AB-2018. 

There is no 3D crystalized molecular structure for HEV hexon protein on which test or analyze the location of these mutations. Although amino acid identities between HAdV-2, HAdV-5, and FAdV-1 range between 47.8–51.36% identity, the overall structure is similar between these viruses consisting on trimers of protein II distributed as three separate “towers” [56]. The location of the mutations was tested on available 3D structures on hexon proteins of HAdV-2, HAdV-3, and FAdV-1 using PYMOL. Both mutations, A231C (aaE77D), and G2598C (aaE866D), were speculated to be located at the bottom of the densely packed pedestal regions (P1, P2) in contact with the penton base found in the capsid interior surface [57,58].

### 3.3. ORF1 Region 

Comparison between all previously published ORF1 sequences [40,41] and those obtained from the current work showed the presence of several unique and shared mutations. A number of mutations (*n* = 39) were detected, of which 17 were synonymous and 22 were non-synonymous (Supplement Table S1). Some mutations were detected in both vaccine sequences (ntG1485A; aaQ495R) and in German sequences obtained from vaccinated flocks suspected to have subclinical HE with increased mortality and higher incidence of Escherichia coli infections (Cases 7, 8, 12, and 17) [41] (ntG1274A; aaI425V) (Supplement Table S1). The phylogenetic tree in Figure 3 shows ORF1 genes of virus 18-1234-AB-2018; 18-0943-AB-2018; and 18-0665-AB-2018 clustering with ORF1 sequences derived from vaccine strains, ORF1 sequences consist of a separate group.

### 3.4. E3 Gene

Eight-point mutations were located in the E3 gene (903 bp), of which five were non-synonymous. Some point mutations were common to many sequences, such as ntC497A (aaP166H), which included most US isolates from Virginia, and ntA517C (aaT173P), which included some vaccine and vaccine-like sequences (Supplement Table S1). The phylogenetic tree in Figure 4a shows sequences 18-1234-AB-2018; 18-0943-AB-2018; and 18-0665-AB-2018 clustering together with vaccine strains, while all the other sequences clustered in a separate group.

### 3.5. Fib knob Domain

**Figure 5:**
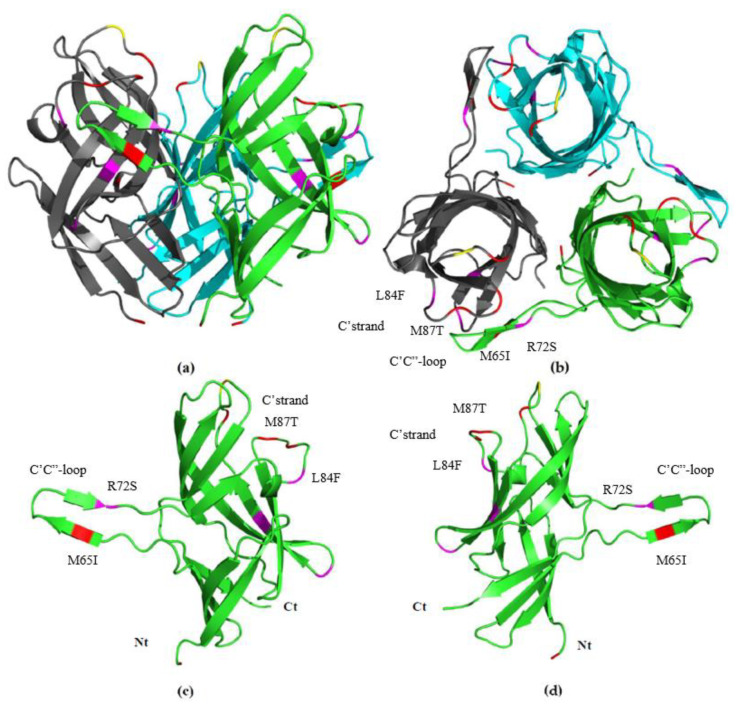
Structure of the HEV *fib* knob domain. Trimeric structure can be seen from side (**a**) and from the top (**b**). Side views of a monomer observed from the outside of the molecule (**c**) and the inside (**d**) with amino-terminus (Nt), and carboxy-terminus (Ct) signaled. Mutations found in HEV Canadian sequences marked in magenta, HEV virulent sequences on red, and one mutation found in an HEV vaccine in yellow. For total list of viruses see Table 3. Figure was constructed following Singh et al. 2015 [4]. M65I and M87T are mutations of Virulent-IL-1998 when compared with an avirulent strain (splenic vaccine) resulting in a 3D change in C’C” loop [4]. R72S, and L84F mutations of sequences 17-0699-BC-2017; 18-0723-BC-2018; and 18-0665-AB-2018 target the same area (C’C”-loop).

Ten point mutations were located in the *fib* knob domain, and similarly to previous research, none of them was silent [40] (Supplement Table S1). None of the four non-synonymous mutations present in the Canadian sequences was shared with previously published strains from US and Israel [40]. These corresponded to: A ntC214A (aaR72S); a ntG252T (aaL84F); a ntG401A (aaG134D); and a ntG414T (aaM138I). The phylogenetic tree in Figure 4b shows sequences 18-1234-AB-2018; and 18-0943-AB-2018 clustering together with vaccine strains, while all the other sequences clustered apart from vaccine strains. There is a 3D crystalized molecular structure for HEV *fib* knob domain on which to analyze the location of these mutations. The amino acid sequences analyzed shared an identity of 97.58–100%. All these mutations can be found in the most exterior part of the domain; three of them were shown to be part of linear loops on the exposed surface of the domain (ntC214A (aaR72S); ntG252T (aaL84F); ntG401A (aaG134D); and the last one, ntG414T (aaM138I), was found to be part of a beta sheet that is also exposed (See Figure 5). Some non-synonymous mutations on the Canadian sequences could be found in the same loop/area than other mutations present in virulent sequences from USA and Israel (See Figure 5).

### 3.6. pTP

Twelve-point mutations, a four-nucleotide change, and a three-nucleotide insertion were located in the pTP gene, from which seven corresponded to non-synonymous mutations at aa296, 362, 460, 521, 522–523, 524, and 529. Interestingly, the non-synonymous mutations corresponding to 521–524 are unique to the commercial vaccines and vaccine-like sequences analyzed (Supplement Table S1).

The sequences included in each of the phylogenetic trees can be observed on Table 3. The single point mutations are given in Supplement Table S1. The sequences studied on this project, in comparison to the H.E. Vac, and Oralvax were found to share: (a) 99.0% to 99.9% nucleotide identity in whole genome sequence (Figure 2a); (b) 99.7% to 100% aa identity in hexon gene (Figure 2b); (c) 99.0% to 99.8% aa identity in the ORF1 gene (Figure 3); (d) 99.3% to 100% aa identity in E3 gene (Figure 4a); and (e) 98.8% to 100% aa identity in the *fib* knob domain (Figure 4b).

### 3.7. Prediction of O-Linked Glycosylation Sites in fib Knob by NetOGlyc Service

Twenty-two aa sequences of *fib* knob domain obtained in this study and previously published were subjected to analysis by the NetOGlyc Server 4.0 service software [59]. This analysis predicted 6 O-glycosylation sites at amino acids 13, 18, 19, 22, 24, and 26 with tight scores within each site in the sequences regarded as vaccine or vaccine-like (Table 3). The same glycosylation spots were located when most of the field strains were analyzed, however higher score variations within each glycosylation areas were found in comparison with the vaccine and vaccine-like sequences. Two sequences, 18-0430, and Virulent-IL-1998, were found to have one extra site at amino acid 10 for a total of seven O-glycosylation sites. Differences with the vaccine profile are marked in red and showed in Table 4.

### 3.8. Prediction of N-Linked Glycosylation Sites in fib Knob by NetNGlyc Service

Twenty-two amino acid sequences of *fib* knob domain obtained in this study and previously published were subjected to analysis by the NetNGlyc Server 1.0 service software [59]. This analysis predicted 10 N-glycosylation sites at amino acids 32, 61, 67, 73, 89, 90, 97, 117, 118, 133, 135, 143, and 148 in the sequences regarded as vaccine or vaccine-like (Table 5). Comment “PRO-X1” on the side of a probable glycosylation site, refers to when a proline is located after an asparagine, deeming highly unlikely that the asparagine get glycosylated due to conformational limitations. The Sequon ASN-XAA-SER/THR comment refers to a sequence of consecutive amino acids that is highly likely to get glycosylated (Table 5). Most of the N-glycosylation areas were located in the remaining strains, but some important differences were observed when compared with most of the vaccine sequences profile: TC Vaccine D had a different agreement at aa143 and aa148, with an extra predicted N-glycosylation site at amino acid 143; Virulent-IL-1998 had a different agreement at aa67, 89, and 90 with an stronger N-glycosylation prediction at aa61; Virulent-US-VA-1996 had a different agreement at aa90 and 148, with one missing glycosylation site at aa89 and a stronger N-glycosylation prediction at aa90; 17-0699, 18-0723, and 18-0665 had a lower agreement at aa67, with one missing glycosylation site at aa67; Virulent-2-US-VA-2005, Virulent-3-US-VA-2005, and Virulent-4-US-VA-2005 had a lower agreement at aa32, and 148, with a weaker glycosylation site prediction at aa32; Virulent-1-US-VA-2005 had a lower agreement at 148; 18-0430 showed a lower agreement at aa135; and 17-0495 and 18-0374 had a lower agreement at aa148 with neither of these sequences having changes in their glycosylation profile when compared with the vaccine profile. Differences with the vaccine profile are marked in red and showed in Table 5.

## 4. Discussion

In the present study, whole genome phylogenetic analysis shows the separation of seven whole genome field sequences into two clusters: (1) the first (Cluster 1) considering vaccine and vaccine-like strains (99.82–99.96% nt similarity), and (2) Cluster 2 (99.71–99.98%), including virulent and suspected-virulent strains together with the splenic vaccine, which is not commercially available in Canada. Similarly, as with previous publications, all major ORFs were located as expected [2,40]. As in previous research [6,40,41], no major changes in either of the genes analyzed were discovered, only single point mutations that may influence the virus ability to cause disease [6,40,41]. 

Recently, several virulence factors of HEV have been identified (i.e., ORF1, E3, and *fib* knob domain) [40]. Although some of the functions of these virulence factors remain to be discovered, there are speculations on their functions. For instance, the protein coded by the ORF1, resembles bacterial sialidases, a group of enzymes that cleave glycosydic linkages of neuraminic acids [60]. These proteins may act as virulence factors for microbial [61,62] and viral infections [63,64]; and may control HEV interactions with host cellular components and, thus, have an effect on pathogenicity and virulence [2]. Interestingly, the product of ORF1 a sialidase, has been recently confirmed as an structural component in the THEV virion, which opens the need for further research on its potential function in the virion [65,66]. Although the E3 gene shares minimal sequence homology with other adeno viruses outside the genus *Siadenovirus*, it seems to resemble the E1A protein of Mastadenoviruses and may code for a transcriptional regulator, this based on the cysteine-rich regions resembling the zinc-binding CR3 domain present in E1A from Mastadenovirus [40,65]. The expression pattern of E3 suggests a possible function in the virus life cycle but its function has not been fully elucidated [65]. The non-synonymous mutations found on the hexon gene are speculated to be in the base of the trimeric hexon protein, perhaps close to the penton with little to no exposure to the host, thus with apparent little importance for antigenicity or pathogenicity. However, according to research conducted on HAdV2 and HAdV5, the area below the protein is important in pH-dependent conformational change of the capsid within the endosome, leading to penetration of the membrane and release of the virus genome into the cytoplasm [54,55,57,58]. Furthermore, upon analysis of 15 HAdV, Crawford–Miksza et al. [57] detected seven hyper variable regions (HVR), from which HVR-1 has a segment that can be found buried in the protein, interacting with the base of the protein. This HAdV HVR-1 has been hypothesized to have an effect on pH-dependent disassembly [55,58]. Further research is needed, including a sound 3D structure imaging of the HEV hexon protein to provide more evidence to this hypothesis. 

The *fib* knob is implicated in the attachment to the host receptor and it is the only adenoviral protein that is glycosylated [40]; thus, an alteration of the amino acid sequence may alter the glycosylation of the protein, resulting in increased infectivity and/or decrease virus neutralization thus modifying the virulence of the virus [40,67]. Interestingly, differences in software-predicted O-linked and N-linked glycosylation areas were witnessed between vaccine/vaccine-like HEV sequences and field HEV sequences; one extra O-linked glycosylation site; and different N-linked profiles. It is worth noting that these software-predicted glycosylation sites would have to be confirmed with relevant techniques, such as liquid chromatography-mass spectrometry (LC-MS/MS) experiments, and that glycosylation patterns vary in different host cell types. Because of the wide different glycosylation scores found in the field HEV sequences, it can be possible that different glycosylation profiles occur between sequences, as some potential sites might be inefficiently glycosylated or miss the chance of post-translational modification [68]. As described before, the proteins relevant for inducing neutralizing antibodies are the hexon protein, and the fiber protein. Although HEV is considered as one serotype, and there is cross-protection between isolates, it is known for some years that there are differences in monoclonal antibodies profiles between avirulent strains (splenic vaccine) and virulent strains (Virulent-IL-1998) [69]. Research in the location of specific antigenic sites for HEV is scarce; however, recent research by Singh et al. 2015, found a crucial difference between the *Fib* knob domain structures of avirulent (splenic vaccine—GenBank AY849321), and virulent virus (Virulent-IL-1998—AF074946). In short, they found that non-synonymous mutations M65I (at the C’C”-loop), and M87T located (C’-strand) were responsible for a difference of 3Å upwards in the C’C”loop, changing the configuration of the protein. In the present work, we found changes on the same area (R72S, and L84F on sequences 17-0699-BC-2017; 18-0723-BC-2018; and 18-0665-AB-2018), as well as others that also may cause further change in the 3-D configuration of the protein, however, further crystallography studies would have to be conducted (Figure 5).

The existence of HEV variants in vaccinated flocks with subclinical infections has also been shown [41]. In agreement with the later study, we showed that turkey farms with recurrent morbidities (e.g., systemic bacterial infections, cellulitis, and elevated mortality), with or without HEV vaccination, have subclinical HEV infections caused by HEV different from vaccine strains. Our objective was to characterize these HEV-positive samples based on whole genome sequencing and/or gene sequences (i.e., hexon, ORF1, E3, and *fib* knob domain). 

Following analysis of previously published virulence factors ORF1, E3, and Fib knob [40] as well as the hexon gene, only two out of nine analyzed sequences were deemed tissue culture vaccine-like strains (one sequence obtained from a vaccinated flock, and the other one from a non-vaccinated flock); while seven out of nine were classified as field strain (three sequences obtained from vaccinated flocks, and the other four from non-vaccinated flocks) (Table 3). Although all flocks present in this study were suspected to have an immunosuppression component, due to a perceived increased susceptibility to secondary bacterial infections [11], it is interesting to note that out of four sequences obtained from HE-vaccinated flocks and with secondary bacterial infections (e.g., cellulitis, systemic bacterial infection, and gangrenous dermatitis) (18-0430; 18-0665; 18-0943; and 18-0988) (Table 1), three were classified as HEV field virus with no recovery of any vaccine sequence. Recovery of a vaccine sequence was expected after successful HE vaccination in these farms as vaccine is expected to reduce clinical signs, but not virus infection (Table 3). The absence of vaccine-like sequences in these vaccinated flocks, and the presence of field sequences may be considered as evidence suggestive of a vaccine failure scenario. Insufficient vaccine-induced immunity or failure to persistently infect the vaccinated turkeys may be due to genomic changes increasing the virulence of field strains that are able to escape the immunity provided by the vaccine or to infect the poults amidst presence of maternal antibodies before vaccination, or perhaps poor vaccine delivery.

Unlike previous research conducted on virulent and avirulent (commercial splenic) sequences [40], in the present work there were five unique amino acid changes conserved in tissue culture vaccine and vaccine-like sequences distributed in two proteins: (1) the hexon protein (1 aa change) and (2) pTP (4 aa mutations). Variations on the hexon gene were only found in one previous paper by Giovanardi, et al. [12] describing insufficient immunity generated by a commercially-available inactivated splenic vaccine used in Italy. The presence of alterations in amino acid sequence in the pTP protein in tissue culture vaccine and vaccine-like sequences may be related to its passage in cell culture system, as the pTP protein is involved in viral replication forming a heterodimer involving AdPol and functions as a protein primer [70]. It is unclear if these aa changes in pTP would influence the replication rate of a given virus under field conditions, but pTP and hexon protein changes can be powerful markers for identifying tissue culture vaccine-like strains. Furthermore, many novel non-synonymous mutations were observed upon analysis of the *Fib*, interestingly, in Canadian field-HEV sequences, these amino acid changes were found in close proximity with other amino acid changes also detected in virulent sequences from Israel and US, suggesting that these mutations are located in functional important locations, perhaps areas targeted by the host immune system. Given that HEV neutralizing antibodies are generated against the hexon and fiber knob proteins, any non-synonymous mutation is potentially important as it may interfere with the induction of immunity induced by vaccines and/or increased virulence of wild type HEV strains [41]. However, it is unclear if these differences in hexon, ORF1, and E3 are responsible for the reported superior immunity strength conferred by splenic vaccines when compared with tissue cultured vaccines [8,36]. It is worth considering that tissue culture vaccines have been manufactured in the 1980s from the Domermuth strain originally used for splenic vaccination since 1970s and that *fib* knob sequences between these strains are identical [35,37,71]. Like previous research on the ORF1 protein, non-synonymous mutations tend to cluster at the terminal portions of ORF1 but in E3, most of the Canadian HEV sequences differ from tissue culture vaccines at location amino acid 173 E3, and a more reduced group of sequences at aa27. These non-synonymous mutations were not only located half way of the E3 protein (aa167, aa146, and aa173) as previous findings [40], but also, at the beginning (aa27) and towards the end of the protein (aa239) which may suggest other biological important areas within the protein. Changes in these two proteins, the sialidase coded by ORF1 and E3, have been hypothesized to modulate virulence by triggering inflammatory shock responses causing intestinal lesions and mortality due to an inability to cause apoptosis [40].

The potential efficacy of a given vaccine is determined by the antigenic similarity of the viruses (vaccine and wild type) involved and the neutralization titer generated by the vaccine towards the wild type virus. In general, double-stranded DNA viruses, such as HEV, have the lowest viral mutation rate (ranging between 2 × 10^−7^ to 9.8 × 10^−8^ substitutions per nucleotide per cell infection) [72,73]. HEV studies have found no major deletions, insertions nor evidence of recombination between viral sequences; grouping of isolates has occurred based on single point mutations discovered within genes of interest such as hexon [12], and mainly ORF1, E3, and *fib* knob domain [40,41]. The main objective of the current study was to characterize HEV-positive spleen samples obtained from clinical cases in turkey flocks in which immunosuppression was suspected, as in the last 10 years there has been an increase in flocks with unusual increased mortality or secondary bacterial infections. These cases were found in turkey meat operations with or without an HEV-vaccination program, which was performed only with commercially available tissue culture HEV vaccines. These vaccines are the only live vaccines authorized in Canada, unlike other parts of North America and Europe which have commercial and autogenous splenic HEV vaccines, as well as HEV inactivated vaccines [6,8,12]. Based on field data recollected by PHS and publications by other researchers [40,41], it can be suggested that field HEV viruses may have acquire adaptive changes perhaps due to vaccine pressure. Thus, the whole genome sequencing of the HEV present in spleens of clinical samples was important to understand the type of virus (if vaccine-related or not) is the main wild type HEV present in these farms. Proper phylogenetic analysis would give the industry insight on this answer and infer virulence given previous research [40,41].

Although the current study yielded valuable data, our samples did not represent the whole meat turkey industry in Canada. We also do not know whether HEV could be recovered from apparently healthy turkey flocks since our focus was to isolate and characterize HEV from clinical samples. 

## 5. Conclusions

The analysis of the HEV sequences have revealed the circulation of field type HEV strains in Canadian turkey flocks with a history of vaccination as well as no vaccination. These strains may be responsible for seroconversion, instead of low-virulent tissue-culture-origin strains. Results suggest that HEVs variability in the field may not be as low as previously thought, as some sequences suggest that some adaptive changes, perhaps caused by an increased vaccine pressure, have occurred and may induce immune evasion (BC strains—*fib* knob domain gene). Finally, as this works shows the circulation of field viruses in vaccinated flocks, and the failure to recover such sequences from clinical samples obtained from vaccinated flocks, a revision/audit of current vaccination practices by the poultry industry is recommended. 

## Figures and Tables

**Table 1:** Details of the samples used in this study in chronological order.

ID	Tissue	Province/Source	Age (Days)	Clinical Case	HE Vaccination Program ^a^	Type of Sequence
H.E. Vac	Vaccine	Arko Labs	N/A	N/A	N/A	Vaccine
Oralvax HE	Vaccine	MSD	N/A	N/A	N/A	Vaccine
17-0495	Spleen	ON	44	↑ Mortality-Surveillance	No	Field
17-0699	Spleen	BC	69	↑ Mortality-Surveillance	No	Field
18-0374	Spleen	AB	52	↑ Mortality-Systemic Bacterial Infections. *Escherichia coli* in Pericardium	No	Field
18-0430	Spleen	AB	110	↑ Mortality-Cellulitis- *Escherichia coli*; *Staphylococcus aureus*; *Enterococcus faecalis*; *Lactobacillus agilis* in Subcutaneous tissue	Yes	Field
18-0665	Spleen	AB	91	↑ Mortality-Systemic Bacterial Infections-*Escherichia coli* in Air Sac and Liver	Yes	Field
18-0723	Spleen	BC	62	↑ Mortality-Surveillance *Escherichia coli* in Air Sac and Liver	No	Field
18-0943	Spleen	AB	61	↑ Mortality-Gangrenous Dermatitis *Escherichia coli*; *Staphylococcus saprophyticus*; *Bacillus pumilus*; *Bacillus altitudinis*; *Staphylococcus chromogenes*; *Staphylococcus chromogenes*; *Clostridium perfringes*; *Staphylococcus lentus* in Subcutaneous tissue	Yes	Vaccine
18-0988	Spleen	AB	117	↑ Mortality-Cellulitis- *Escherichia coli*; *Staphylococcus aureus*; *Enterococcus* in Subcutaneous tissue	Yes	Field
18-1234	Spleen	AB	77	↑ Mortality-Systemic Bacterial Infections- *Escherichia coli* in Pericardium and Air Sac	No	Vaccine

^a^ Hemorrhagic enteritis (HE)-vaccination program refers to using a full dose of vaccine (≥10^2.6^ TCID_50_) between 3.5–6 weeks of age, or twice using a lower dose (e.g., 2/3 of a dose or ≥10^2.4^ TCID_50_) at days 25 and 35. ↑ Increased.

**Table 2:** Primer sequences used to amplify the HEV genome and open reading frame (ORF)1 gene. N/A refers to a sequence that was design for this study and not obtained from another publication. Genome positions refer to the splenic vaccine strain (GenBank accession # AY849321.1).

Name	Sequence	Target	Reference	Position	Amplicon	Annealing T	Extension
THEV-Whole-F1	ATGCTTGGGAGGGGATTTCG	THEV	This study	21–40	26,129	59 °C	14 min
THEV-Whole-R1	AACCGGAAAAGAAGGCGGAT	THEV	This study	26,131–26,150			
Alkie-HEV-ORF1-F1	CTGACCTTGTCGTCCGTGC	ORF1	[41]	283–301	1537	62 °C	2 min
HEV3’For-951	TGGCGGCAATGGCTTAGTAA	ORF1	This study	951–970			
Alkie-HEV3’Rev	GGATACAATTGACCATTGGAAG	ORF1	[41]	1799–1820			
THEV-E3-Fw	CTCCCCTAGTCACCTGACCA	E3	This study	20,738–20,757	1807	59 °C	2 min
THEV-E3-Rv	AACGCTTTCCAGGAGTAGCC	E3	This study	22,525–22,544			
THEV-Fib-Fw	GGCTACTCCTGGAAAGCGTT	Fib	This study	22,525–22,544	2021	59 °C	2.5 min
THEV-Fib-Rv	GTCAGCTTGCAACCACCAAG	Fib	This study	24,550–24,569			
THEV-Fib-Fw	GGCTACTCCTGGAAAGCGTT	Fib	This study	22,525–22,544	1502	59 °C	2 min
THEV-Fib-Rv2	GCGCACCTGCAAAGTCAAAT	Fib	This study	24,007–24,026			

**Table 3:** List of all HEV sequences in the study with GenBank accession numbers.

Sequence ^a^	Phylogenetic Tree					GenBank Number	Genome Size (nt)	Paper Published
	Whole Genome	Hexon	ORF1	E3	*fib* Knob Domain			
H.E.Vac	X	X	X	X	X	MT603863 MT603864 MT603862 MT603869 MT603871 MT603861 MT603865 MT603870 MT603866 MT603867 MT603868	26,289	This study
Oralvax HE	X	X	X	X	X		26,270	
17-0495-ON-2017			X	X	X		3850	
17-0699-BC-2017	X	X		X	X		26,115	
18-0374-AB-2018	X	X		X	X		25,997	
18-0430-AB-2018			X	X	X		3850	
18-0665-AB-2018	X	X		X	X		25,717	
18-0723-BC-2018	X	X		X	X		26,115	
18-0943-AB-2018	X	X		X	X		26,289	
18-0988-AB-2018	X	X		X	X		26,100	
18-1234-AB-2018	X	X		X	X		26,120	
Virulent-IL-1998 ^b^	X	X	X	X	X	AF074946	26,263	[2]
Splenic Vaccine	X	X	X	X	X	AY849321	26,266	[40]
Virulent-US-VA-1996 ^b^			X	X	X	DQ868929	3857	
Virulent1-US-VA-2005 ^b^			X	X	X	DQ868931	3857	
Virulent2-US-VA-2005 ^b^			X	X	X	DQ868932	3857	
Virulent3-US-VA-2005 ^b^			X	X	X	DQ868933	3857	
Virulent4-US-VA-2005 ^b^			X	X	X	DQ868934	3857	
Marble spleen vaccine			X	X	X	DQ868930	3857	
Tissue culture vaccine A			X	X	X	DQ868935	3857	
Tissue culture vaccine B			X	X	X	DQ868936	3857	
Tissue culture vaccine C			X	X	X	DQ868937	3857	
Tissue culture vaccine D			X	X	X	DQ868938	3857	
Case1-DE-1989			X			KX944266	1481	[41]
Case2-DE-2010			X			KX944267	1388	
Case3-DE-2012			X			KX944268	1433	
Case4-DE-2008			X			KX944269	1412	
Case5-DE-2008			X			KX944270	1296	
Case6-DE-2008			X			KX944271	1385	
Case7-DE-2008			X			KX944272	1492	
Case8-DE-2008			X			KX944273	1492	
Case9-DE-UNK			X			KX944274	1326	
Case10-DE-2008			X			KX944275	1326	
Case11-DE-2008			X			KX944276	1326	
Case12-DE-2008			X			KX944277	1493	
Case13-DE-2012			X			KX944278	1274	
Case14-DE-2012			X			KX944279	1265	
Case15-DE-2012			X			KX944280	1265	
Case16-DE-2008			X			KX944281	1385	
Case17-DE-2012			X			KX944282	1500	

^a^ Origin of the strain in name. IL—Israel; DE—Germany; US-VA—United States Virginia; AB—Canada, Alberta; BC—Canada, British Columbia; ON—Canada, Ontario. ^b^ Sequences defined as “Virulent”: Were proven to cause hemorrhagic enteritis when inoculated in susceptible turkeys in the relevant publication.

**Table 4:** List of 22 *fib* knob sequences and their corresponding NetOGlyc 4.0 Server prediction results (threshold score ≥ 0.5).

ID	Type of Sequence	O-Glycosylation Site	Score	O-Glyc Results
H.E. Vac Oralvax HE TC Vaccine A TC Vaccine B TC Vaccine C TC Vaccine D Marble Spleen Vaccine Splenic Vaccine 18-1234 18-0943	Vaccine	13	0.76–0.77	Positive-6 locations
		18	0.63	
		19	0.61	
		22	0.54	
		24	0.65	
		26	0.71	
17-0495 17-0699 18-0374 18-0665 18-0723 18-0988 Virulent-US-VA-2005 Virulent-1-US-VA-2005 Virulent-2-US-VA-2005 Virulent-3-US-VA-2005 Virulent-4-US-VA-2005	Field	13	0.76–0.77	Positive-6 locations
		18	0.62–0.66	
		19	0.59–0.61	
		22	0.53–0.59	
		24	0.62–0.66	
		26	0.65–0.72	
18-0430 Virulent-IL-1998	Field	10	0.5	Positive-7 locations
		13	0.77	
		18	0.63–0.64	
		19	0.62	
		22	0.54–0.55	
		24	0.66	
		26	0.71–0.72	

**Table 5:** List of 22 Fib knob sequences and their corresponding NetNGlyc 1.0 Server prediction results (threshold score ≥ 0.5).

ID	Type of Sequence	N-Glycosylation Site	Potential	Agreement	N-Glyc Results ^c^	Comments
H.E. Vac Oralvax HE TC Vaccine A TC Vaccine B TC Vaccine C Marble Spleen Vac. Splenic Vaccine 18-1234 18-0943 18-0988	Vaccine Vaccine Vaccine Vaccine Vaccine Vaccine Vaccine Vaccine Vaccine Field	32-NGQF	0.6786	(9/9)	++	
		61-NIGV	0.7398	(9/9)	++	
		67-NPTF	0.5111	(6/9)	+	PRO-X1 ^a^. Sequon ASN-XAA-SER/THR ^b^
		73-NKSI	0.6818	(9/9)	++	Sequon ASN-XAA-SER/THR
		89-NNTY	0.6219	(8/9)	+	
		90-NTYI	0.6121	(8/9)	+	
		97-NGGV	0.6647	(9/9)	++	
		117-NNSS	0.5110	(5/9)	+	Sequon ASN-XAA-SER/THR
		118-NSSF	0.4376	(7/9)	-	
		133-NGNP	0.1234	(9/9)	---	
		135-NPHM	0.5491	(7/9)	+	PRO-X1
		143-NPVP	0.1229	(9/9)	---	
		148-NIKM	0.6002	(8/9)	+	
TC Vaccine D	Vaccine	32-NGQF	0.6784	(9/9)	++	
		61-NIGV	0.7398	(9/9)	++	
		67-NPTF	0.5111	(6/9)	+	PRO-X1 ^a^. Sequon ASN-XAA-SER/THR ^b^
		73-NKSI	0.6818	(9/9)	++	Sequon ASN-XAA-SER/THR
		89-NNTY	0.6219	(8/9)	+	
		90-NTYI	0.6121	(8/9)	+	
		97-NGGV	0.6648	(9/9)	++	
		117-NNSS	0.5110	(5/9)	+	Sequon ASN-XAA-SER/THR
		118-NSSF	0.4377	(7/9)	-	
		133-NGNP	0.1234	(9/9)	---	
		135-NPHM	0.5493	(7/9)	+	PRO-X1
		143-NPVS	0.5489	**(6/9)**	**+**	
		148-NIKM	0.5516	**(6/9)**	+	
Virulent-IL-1998	Field	32-NGQF	0.6786	(9/9)	++	
		61-NIGV	0.7589	(9/9)	**+++**	
		67-NPTF	0.5039	**(4/9)**	**+**	PRO-X1 ^a^. Sequon ASN-XAA-SER/THR ^b^
		73-NKSI	0.6810	(9/9)	++	Sequon ASN-XAA-SER/THR
		89-NNTY	0.6149	**(7/9)**	+	
		90-NTYI	0.5604	**(7/9)**	+	
		97-NGGV	0.6506	(9/9)	++	
		117-NNSS	0.5112	(5/9)	+	Sequon ASN-XAA-SER/THR
		118-NSSF	0.4377	(7/9)	-	
		133-NGNP	0.1233	(9/9)	---	
		135-NPHM	0.5494	(7/9)	+	PRO-X1
		143-NPVP	0.1229	(9/9)	---	
		148-NIKM	0.6004	(8/9)	+	
Virulent-US-VA-1996	Field	32-NGQF	0.6786	(9/9)	++	
		61-NIGV	0.7400	(9/9)	++	
		67-NPTF	0.5110	(6/9)	+	PRO-X1 ^a^. Sequon ASN-XAA-SER/THR ^b^
		73-NKSI	0.6818	(9/9)	++	Sequon ASN-XAA-SER/THR ^b^
		90-NTYI	0.6771	**(9/9)**	**++**	
		97-NGGV	0.6619	(9/9)	++	
		117-NNSS	0.5109	(5/9)	+	Sequon ASN-XAA-SER/THR
		118-NSSF	0.4378	(7/9)	-	
		133-NGNP	0.1234	(9/9)	---	
		135-NPHM	0.5493	(7/9)	+	PRO-X1
		143-NPVP	0.1229	(9/9)	---	
		148-NIKM	0.6001	**(7/9)**	+	
18-0430	Field	32-NGQF	0.6786	(9/9)	++	
		61-NIGV	0.7399	(9/9)	++	
		67-NPTF	0.5111	(6/9)	+	PRO-X1 ^a^. Sequon ASN-XAA-SER/THR ^b^
		73-NKSI	0.6817	(9/9)	++	Sequon ASN-XAA-SER/THR ^b^
		89-NNTY	0.6220	(8/9)	+	
		90-NTYI	0.6122	(8/9)	+	
		97-NGGV	0.6647	(9/9)	++	
		117-NNSS	0.5111	(5/9)	+	Sequon ASN-XAA-SER/THR
		118-NSSF	0.4379	(7/9)	-	
		133-NGNP	0.1278	(9/9)	---	
		135-NPHI	0.5884	**(6/9)**	+	PRO-X1
		143-NPVP	0.1086	(9/9)	---	
		148-NIKM	0.5840	(8/9)	+	
17-0495 18-0374	Field Field	32-NGQF	0.6786	(9/9)	++	
		61-NIGV	0.7398	(9/9)	++	
		67-NPTF	0.5110	(6/9)	+	PRO-X1 ^a^. Sequon ASN-XAA-SER/THR ^b^
		73-NKSI	0.6818	(9/9)	++	Sequon ASN-XAA-SER/THR ^b^
		89-NNTY	0.6219	(8/9)	+	
		90-NTYI	0.6120	(8/9)	+	
		97-NGGV	0.6647	(9/9)	++	
		117-NNSS	0.5108	(5/9)	+	Sequon ASN-XAA-SER/THR
		118-NSSF	0.4378	(7/9)	-	
		133-NDNP	0.1003	(9/9)	---	
		135-NPHM	0.5519	(7/9)	+	PRO-X1
		143-NPVP	0.1249	(9/9)	---	
		148-NIKM	0.6000	**(7/9)**	+	
17-0699 18-0723 18-0665	Field Field Field	32-NGQF	0.6786	(9/9)	++	
		61-NIGV	0.7398	(9/9)	++	
		67-NPTF	0.4794	**(4/9)**	**-**	Sequon ASN-XAA-SER/THR ^b^
		73-NKSI	0.7094	(9/9)	++	
		89-NNTY	0.6257	(8/9)	+	
		90-NTYI	0.6410	(8/9)	+	
		97-NGGV	0.6649	(9/9)	++	
		117-NNSS	0.5111	(5/9)	+	Sequon ASN-XAA-SER/THR
		118-NSSF	0.4378	(7/9)	-	
		133-NGNP	0.1234	(9/9)	---	
		135-NPHM	0.5494	(7/9)	+	PRO-X1
		143-NPVP	0.1229	(9/9)	---	
		148-NIKM	0.5999	(7/9)	+	
Virulent-1-US-VA-2005	Field	32-NGQF	0.6773	(9/9)	++	
		61-NIGV	0.7398	(9/9)	++	
		67-NPTF	0.5110	(6/9)	+	PRO-X1 ^a^. Sequon ASN-XAA-SER/THR ^b^
		73-NKSI	0.6818	(9/9)	++	Sequon ASN-XAA-SER/THR ^b^
		89-NNTY	0.6219	(8/9)	+	
		90-NTYI	0.6120	(8/9)	+	
		97-NGGV	0.6647	(9/9)	++	
		117-NNSS	0.5108	(5/9)	+	Sequon ASN-XAA-SER/THR
		118-NSSF	0.4378	(7/9)	-	
		133-NGNP	0.1234	(9/9)	---	
		135-NPHM	0.5492	(7/9)	+	PRO-X1
		143-NPVP	0.1229	(9/9)	---	
		148-NIKM	0.6000	**(7/9)**	+	
Virulent-2-US-VA-2005 Virulent-3-US-VA-2005 Virulent-4-US-VA-2005	Field Field Field	32-NGQF	0.6771	**(8/9)**	**+**	
		61-NIGV	0.7397	(9/9)	++	
		67-NPTF	0.5108	(6/9)	+	PRO-X1 ^a^. Sequon ASN-XAA-SER/THR ^b^
		73-NKSI	0.6817	(9/9)	++	Sequon ASN-XAA-SER/THR ^b^
		89-NNTY	0.6219	(8/9)	+	
		90-NTYI	0.6121	(8/9)	+	
		97-NGGV	0.6646	(9/9)	++	
		117-NNSS	0.5107	(5/9)	+	Sequon ASN-XAA-SER/THR
		118-NSSF	0.4378	(7/9)	-	
		133-NGNP	0.1234	(9/9)	---	
		135-NPHM	0.5591	(7/9)	+	PRO-X1
		143-NHVP	0.1038	(9/9)	---	
		148-NIKM	0.5943	**(6/9)**	+	

^a^ Pro-X1: When a Pro residue is located immediately after an Asn residue. Most likely the Asn is not glycosylated due to conformational limitations. ^b^ Sequon ASN-XAA-SER/THR indicates a sequence of consecutive amino acids where a polysaccharide can attach. ^c^ N-Glyc results: Any potential location crossing the threshold of 0.5 would represent a predicted glycosylated site. Potential of N-glycosylation predicted site is predicted as low “+” or strong “++”, “+++” potential; whereas scores with “-”, “--”, and “---” indicate that the site is most likely not glycosylated. Glycosylation pattern different from H.E. Vac and Oralvax HE, are **underlined in bold**.

## Supplementary Materials

The following are available online at https://www.mdpi.com/1999-4915/12/9/941/s1, Table S1: Sequence differences in hexon, ORF1, E3, and *fib* knob domain.
