# Supplementary material for: Molecular Characterization of Hemorrhagic Enteritis Virus (HEV) Obtained from Clinical Samples in Western Canada 2017–2018
Source: Viruses. 2020 Aug 26;12(9):941. doi: 10.3390/v12090941 (PMC7551992; doi:10.3390/v12090941)
Supplement: Supplementary file 1 [file viruses-12-00941-s001.pdf]

Supplement Table S1. Sequence differences in Hexon, ORF1, E3, and *fib* knob domain. X##Y; X corresponds to consensus nucleotide/amino acid, ## to position, and Y to the changing nucleotide/amino acid.

Nt Mutation	Aa Mutation	Gene	Sequences affected
A82G	I28V	ORF1	18-0374-AB-2018; 17-0495-ON-2018;
G84A	I28M		Virulent-1-US-VA-2005; Virulent-2-US-VA-2005; Virulent-3-US-VA-2005; Virulent-4-US-VA-2005; Marble Spleen Vaccine.
G100C	A34P		Case2-DE-2010
G122T	G41V		Case2-DE-2010
-147T	NA		Virulent-IL-1998
A161C	I54L		Case2-DE-2010
G176C	E59Q		Case2-DE-2010
C216T	A72V		Case2-DE-2010
C246G	A82G		Virulent-US-VA-1996
TT267-268GC	V89G		Case2-DE-2010
C297T	A99V		Case2-DE-2010
G353A	V118I		Case2-DE-2010
T416C	P139S		Case2-DE-2010.
T455K	L152V/L		18-0374-ON-2018
T522C	V174A		Case2-DE-2010
T525Y	V175A/V		Oralvax-HE; H.E.Vac
T525C	V175A		TC Vaccine D
G603C	S201T		Case2-DE-2010
A1157G	T386A		18-0374-ON-2018; 17-0495-ON-2017; 18-0988-AB-2018; 18-0430-AB-2018
A1274G	I425V		Virulent-IL-1998; Case1-DE-1989; Case2-DE-2010; Splenic Vaccine; 18-0988-AB-2018; 18-0430-AB-2018; 18-0374-AB-2018; 17-0495-ON-2017; 17-0699-BC-2017; 18-0723-BC-2018; Splenic Vaccine; Virulent-US-VA-1996; Virulent1-US-VA-2005; Virulent2-US-VA-2005; Virulent3-US-VA-2005; Virulent4-US-VA-2005; Marble Spleen Vaccine.
A1420C	Q473H		Splenic Vaccine
A1485G	Q495R		Oralvax-HE; H.E.Vac; TC Vaccine A-B-C-D; Case12-DE-2008; Case8-DE-2008; Case7-DE-2008; Case17-DE-2008; 18-1234-AB-2018; 18-0943-AB-2018; 18-0665-AB-2018
G1534T	L511F		Virulent-US-VA-1996
C235G	H79D	Iva2 (RC)*	Virulent-IL-1998
G433A	D145N		Virulent-IL-1998
G490T	D164Y		18-0665
C316T	H106Y	AdPol (Reverse Complement	Virulent-IL-1998
G800A	R267Q		17-0699; 18-0723; 18-0665
A1259G	N420S		17-0699; 18-0723
G1640C	C547S		Virulent-IL-1998
C2092A	P698T		18-0665
G2312A	R771K		18-0723
G2566A	D856N		18-0988
T3004A	Y1002N		Virulent-IL-1998
G3097A	E1033K		Splenic Vaccine
T888G	N296K	pTP	18-0665-AB-2018
G1085A	G362E		18-0665-AB-2018; 18-0374-ON-2018; 17-0699-BC-2017; 18-0723-BC-2018
A1378G	I460V		18-0988-AB-2018
ACTC1561-1564CAAG	T521Q		Oralvax-HE; H.E.Vac; 18-1234-AB-2018; 18-0943-AB-2018.
---1566-1568ACA	Q522-523EQ		Oralvax-HE; H.E.Vac; 18-1234-AB-2018; 18-0943-AB-2018.
C1571A	A524E		Oralvax-HE; H.E.Vac; 18-1234-AB-2018; 18-0943-AB-2018.
C1585G	L529V		Splenic Vaccine
C217G	Q73E	pVII	Splenic Vaccine

C286T	P96S		18-1234
A231C	E77D	Hexon	18-0665-AB-2018
G2598C	E866D	Hexon	Oralvax-HE; H.E.Vac; 18-1234-AB-2018; 18-0943-AB-2018.
C30G	D10E	DBP (RC)	18-0723
G193T	A65S	100K	18-0988
C224G	A75G	100K	18-0723; 18-0374; 18-0665; 17-0699
A414C	K138N	100K	18-0723; 18-0374; 18-0665; 17-0699
C665T	T222I	100K	18-0723
G1249A	T417A	100K	18-0374
G1792A	V598I	100K	18-0988
A354C	K118N	33K	18-0665
C80T	A27V	E3	18-0430-AB-2018; 18-0988-AB-2018
A437C	K146T	E3	Virulent-IL-1998
C497A	P166H	E3	Virulent1-US-VA-2005; Virulent2-US-VA-2005; Virulent3-US-VA-2005; Virulent4-US-VA-2005.
A517C	T173P	E3	TC Vaccine A-B-C-D; Oralvax-HE; H.E. Vac; 18-1234-AB-2018; 18-0943-AB-2018; 18-0665-AB-2018.
G717C	K239N	E3	Splenic Vaccine
T149G	V51G	Fiber	18-0665
C350T	T118I	Fiber	17-0699; 18-0723; 18-0374
G859A	V288I	Fiber	18-1234
G1062A	M355I	Fiber	Virulent-IL-1998
C1081A	R362S	Fiber	17-0699; 18-0665; 18-0723
G1119T	L374F	Fiber	17-0699; 18-0665; 18-0723
T1127C	M377T	Fiber	Virulent-IL-1998
G83A(1268)	G28D (424)	*fib* knob domain (Part of Fiber gene)	Virulent1-US-VA-2005; Virulent2-US-VA-2005; Virulent3-US-VA-2005; Virulent4-US-VA-2005.
G195A(1380)	M65I (461)	*fib* knob domain (Part of Fiber gene)	Virulent-IL-1998
C214A(1399)	R72S (468)	*fib* knob domain (Part of Fiber gene)	18-0723-BC-2018; 18-0665-AB-2018; 17-0699-BC-2017
G252T(1437)	L84F (480)	*fib* knob domain (Part of Fiber gene)	18-0723-BC-2018; 18-0665-AB-2018; 17-0699-BC-2017;
T260C(1445)	M87T (483)	*fib* knob domain (Part of Fiber gene)	Virulent-IL-1998
A265G(1450)	N89D (485)	*fib* knob domain (Part of Fiber gene)	Virulent-US-VA-1996
G401A(1586)	G134D(530)	*fib* knob domain (Part of Fiber gene)	18-0374-ON-2018; 17-0495-ON-2017
G414T(1599)	M138I(534)	*fib* knob domain (Part of Fiber gene)	18-0374-ON-2018
C431A(1616)	P144H(540)	*fib* knob domain (Part of Fiber gene)	Virulent2-US-VA-2005; Virulent3-US-VA-2005; Virulent4-US-VA-2005
C436T(1621)	P146S(542)	*fib* knob domain (Part of Fiber gene)	TC Vaccine D
C152T	P51L	ORF7	17-0699; 18-0374; 18-0665; 18-0723

* RC-Reverse Complement
